# Effectiveness of selective serotonin reuptake inhibitors in inpatients with selective mutism compared to social anxiety disorder: an observational study

**DOI:** 10.1007/s00787-025-02950-z

**Published:** 2026-01-16

**Authors:** Charlotte Sachs, Simeon Platte, Andreas G. Chiocchetti, Christine M. Freitag

**Affiliations:** Department of Psychiatry, Psychosomatics and Psychotherapy of Childhood and Adolescence, University Hospital Frankfurt, Goethe Universität, Frankfurt Am Main, Germany

**Keywords:** Selective mutism, Social anxiety disorder, SSRI, Speaking pattern, Duration of treatment

## Abstract

Selective Mutism (SM) is a rare childhood anxiety disorder characterized by a persistent inability to speak in specific social settings despite adequate speaking ability in others, sharing etiological and clinical overlap with Social Anxiety Disorder (SAD). While evidence for pharmacological treatment in SM is limited, selective serotonin reuptake inhibitors (SSRIs) have demonstrated potential efficacy, but studies on effectiveness in routine clinical practice are lacking. This single-center, retrospective observational study analysed data from 2011 to 2021 on children aged 4–14 years with SM or SAD treated as inpatients at a German university hospital. The mean age at admission was 11.9 years in both groups. Using propensity score matching and statistical modelling, we compared SSRI prescription patterns, dosage, and duration of inpatient/day-care treatment (DOT) between groups. In the SM group, we additionally tested for differences in speaking pattern at discharge comparing children with and without SSRI medication. SSRI treatment was more frequent in SM (75.4%) than in SAD (55.0%, *p* = 0.011). Adjusted for pattern of additional internalizing, externalizing and specific developmental disorders, no main effects of group (SM versus SAD), nor of SSRI treatment or dosage between and across groups were found on DOT. In the SM group, SSRI treatment was associated with more strongly improved speaking pattern at discharge compared to treatment without SSRI (*p* = 0.009). Results are discussed in relation to optimal dosage and effectiveness of SSRI treatment on improved speaking pattern in SM.

## Introduction

Selective Mutism (SM) is a rare mental disorder with a typical before the age of 5 and often follows a chronic course [1, 2]. According to DSM-5 [3], SM is classified as anxiety disorder, characterized by a continuous lack of speech in specific social situations when speaking is expected or required, despite the child’s ability to speak in other settings. Affected children and adolescents show age appropriate language development, and are able to verbally communicate with familiar partners. They speak fluently with family members or close friends, yet stay silent during other interactions, such as at school, or during interaction with strangers. The selective speaking has persisted for at least one month, excluding the first month of school, and is not better explained by other mental or neurodevelopmental disorders, such as autism spectrum (ASD) or psychotic disorders. ICD-11 [4] similarly to DSM-5 classifies SM as anxiety disorder with comparable diagnostic criteria. SM has a lifetime prevalence of 0.2% to 2%, with higher rates in bi-/multilingual families, and affects girls about twice as often as boys [2, 5, 6].

The aetiology of SM has rarely been studied, with genetic and environmental factors likely contributing to individual risk and onset. Several studies identified overlapping aspects between SM and social anxiety disorder (SAD) regarding aetiology and psychopathology. Both disorders are associated with predisposing factors such as behavioural inhibition (BI) during early childhood [7–9]. Retrospective studies have observed even stronger BI in individuals with SM than in those with other anxiety disorders [7, 10]. Clinical observations report shared features of SM and SAD, including speaking pattern [10] or fear-related cognitions [11]. Children with SM or SAD tend to experience higher levels of anxiety, fewer friendships, and greater difficulties forming social relationships compared to typically developing peers [10]. Additionally, SAD frequently co-occurs in individuals with SM [9]. This substantial overlap has led to SM being described as an extreme form of SAD [12]. 

Regarding evidence-based interventions for SM, hardly any randomized-controlled trials (RCTs) on psychopharmacological interventions have been conducted [13], whereas recent reviews specifically highlighted the need for those [14, 15]. Selective serotonin reuptake inhibitors (SSRIs) have been studied by a limited number of small RCTs. Their potential efficacy in SM has been delineated from the aetiopathological overlap with SAD, and the established efficacy of SSRIs in the treatment of anxiety disorders in children and youths, specifically for SAD [16–18]. A positive dose–response relationship has been observed for adults treated with SSRIs regarding anxiety-related behavioural and cognitive outcomes [19]. Transferring these findings, several case reports and small RCTs have explored the effect of SSRIs on SM. For instance, one case report documented fluoxetine use (initial dose: 4 mg/day, final dose: 8 mg/day) in a 4-year-old girl, showing marked improvement of speaking behaviour within five days, and free speech within 20 days [20]. Similarly, in an 8-year-old girl, seven months of fluoxetine treatment (10 mg/day) [21] led to a reduction in mutism symptoms as measured by the Global assessment of functioning scale (GAF, reduction of 21 points). Fluoxetine was further studied in a 9-week open trial, involving 21 children (mean age: 8.2 years (SD 2.6), age range 5 to 14, mean fluoxetine dose: 28.1 mg/day, range: 10–60 mg) [22]. The study described symptom improvement in 16 (76%) participants with SM, i.e. diminished anxiety and more frequent speech in public settings, with no response observed in individuals receiving less than 20 mg/day. A double-blind, placebo-controlled trial in 15 children with SM (mean age: 8.5 years (SD 1.9), age range 6 to 11) over 12 weeks, found fluoxetine (mean dose: 21.4 mg/day, range: 12–27 mg/day) superior to placebo in reducing anxiety and mutism symptoms [23]. Sertraline has also been investigated in a low-quality 16-week double-blind trial with five children (mean age: 6.8 years (SD 2.5), age range 5 to 11). Participants alternated between no medication, placebo, or sertraline (maximum dosage 100 mg/day). Improvements of selective speech, shyness and anxiety were noted during sertraline treatment, assessed by a Parent Goal Attainment Scale (GAS), and the Child Behavior Checklist (CBCL) [24]. Regarding the treatment of SM with citalopram, only case series have been conducted so far, which did not yield statistically significant results. However, citalopram showed positive effects [15, 25, 25, 26]. Despite the lack of robust evidence, SSRIs remain the pharmacological intervention of choice for SM in clinical practice and are frequently prescribed as part of multimodal treatment approaches.

Over the last years, several well-designed studies have provided empirical support for psychotherapeutic interventions in the treatment of SM. Cognitive-behavioural therapy (CBT), often adapted to the specific features of SM, has shown promising outcomes in both case series and controlled trials. For instance, structured (group-) treatment programs focusing on gradual exposure, reinforcement strategies, and parental involvement have demonstrated efficacy in reducing mutism symptoms and improving speaking behaviour in school and other social settings [27–29]. Oerbeck et al. showed significant improvement throughout a psychotherapeutic intervention (*p* = 0.004) on mutistic symptoms, especially in younger participants [30]. These findings have strengthened the evidence base for behavioural treatments and have led to their recommendation in current clinical guidelines.

Given the scarcity of studies on SSRI use in children and youths with SM, this retrospective study aimed to evaluate SSRI effectiveness by comparing individuals with SM to those with SAD, treated as day-care or inpatients at a large university-based Department of Child and Adolescent Psychiatry, Psychosomatics, and Psychotherapy in Germany. Although behavioural and cognitive-behavioural interventions have demonstrated promising effects in the treatment of SM, systematic research on pharmacological approaches remains limited. This retrospective study therefore investigated SSRI prescription patterns — defined as the type of SSRI substance (active agent), prescribed dosage, and frequency of administration — across both diagnostic groups, as well as the outcomes of multimodal treatment, specifically measured as (a) duration of inpatient/day-care treatment (DOT) and (b) clinical improvement in speaking behaviour among individuals with SM. In the absence of prior comparative studies, SSRI prescription patterns were analysed in an exploratory manner. We hypothesized that SSRI treatment would be associated with a shorter DOT for both groups compared to multimodal therapy without SSRI use. Additionally, we expected that individuals with SM receiving SSRI treatment would demonstrate greater improvements in speaking behaviour compared to those not treated with SSRIs.

## Material and methods

The study is a single-centre non-interventional retrospective study conducted at Goethe University, University Hospital Frankfurt, Department of Child and Adolescent Psychiatry, Psychosomatics and Psychotherapy.

### Study population, data source and extracted variables

#### Inclusion criteria

Children and adolescents aged 4.0 to 14.0 years, meeting ICD-10 criteria [31] for SM or SAD at discharge; first day-care or inpatient treatment between 2011/12/01–2021/11/30 (excluded: prior emergency admissions for crisis intervention). The study population was divided into two groups, namely SM and SAD, whereas the SAD group was matched to the SM group. Inclusion criteria for the SM group: meeting ICD-10 criteria for SM (F94.0) as primary or secondary diagnosis. Inclusion criteria for the SAD group: meeting ICD-10 criteria for social anxiety disorder (F40.1) or social anxiety disorder of childhood (F93.2) as primary or secondary diagnosis. If patients met ICD-10 criteria for both SM and SAD, they were assigned to the SM group.

#### Exclusion criteria for both groups

Any diagnosis of a mental or behavioural disorder due to psychoactive substance use (ICD-10 F10-F19), schizophrenia, schizotypal and delusional disorders (ICD-10 F20-F29), manic episodes (ICD-10 F30), bipolar affective disorder (ICD-10 F31), cyclothymia (ICD-10 F34.0), dissociative (conversion) disorders (ICD-10 F44), eating disorders (ICD-10 F50), disorders of adult personality and behaviour (ICD-10 F60-F69, except ICD-10 F63.8), intellectual disability (ICD-10 F70-F79), pervasive developmental disorder (ICD-10 F84); premature discontinuation of treatment, discharge against medical advice, SSRI medication prior to admission (ensuring consistency across treatment comparisons).

#### Multimodal intervention

Both, day-care and inpatient wards follow an almost identical treatment approach and were therefore integrated into a unified treatment setting for analysis. Treatment is provided by a multi-professional team of child and adolescent psychiatrists, psychological psychotherapists, nurses, childcare workers, speech and language as well as occupational and physio-therapists. Interventions include individual and group-based CBT for patients and parents as well as pharmacotherapy, occupational therapy, physiotherapy, and speech and language therapy for the child. Interventions are aligned with current scientific knowledge and applicable guidelines. All patients, regardless of diagnosis, receive evidence-based CBT as part of standard care. However, treatment plans are tailored to individual clinical needs. For children with SM, this includes disorder-specific therapeutic elements in accordance with treatment guidelines, such as targeted exposure-based interventions to reduce anxiety and the inclusion of speech and language therapy, which is not routinely provided for other diagnostic groups (e.g., patients with depression only). In 2021, the average length of stay in child and adolescent psychiatric facilities in Germany was around 33 days [32]. This figure encompasses both, inpatient and day-care treatment on longer-term therapeutic wards, as well as emergency crisis interventions, some of which may last only a few hours or days [33]. Compared internationally, Germany provides a higher amount of day-care and inpatient mental health facilities than most countries [34].

#### Data collection and processing

Retrospective data, routinely collected during the treatment, were extracted from electronic medical records (software: Orbis by Dedalus) and pre-specified in alignment with the study aims. Data were analysed and published in a pseudonymised form, with patient identification numbers (consistent across recurring treatments) and case identification numbers (unique to each treatment interval) generated to distinguish between first-time admissions and readmissions. Treatments interrupted by temporary discharges, such as those due to mandatory COVID-19 mitigation regulations, and followed by a consecutive readmission within 28 days were considered cumulatively as one treatment period. The respective cases (using case identification numbers) were consolidated through a merging process to ensure accurate data integration and alignment. For a treatment period to qualify as day-care or inpatient treatment, a minimum duration of 30 days was required.

The following data were extracted or calculated for each individual:

- age at admission (years)
- sex (assigned at birth)
- dates of admission and discharge
- length of treatment (days)
- results of psychometric testing – manually extracted from the discharge letter
- initial and pre-discharge measured bodyweight and body height
- ICD-10 diagnoses (chapter 5, F10-F99) at discharge
- for the SM group: speaking behaviour at discharge (see below)
- prescribed pharmacological compounds (see below)

All ICD-10 diagnoses were based on a standardized diagnostic procedure routinely implemented in the clinical setting. This procedure includes clinical interviews with the child and caregivers, behavioural observations, and standardized psychological assessments using validated questionnaires. Diagnoses were made by trained child and adolescent psychiatrist or psychotherapists, based on discussions with the entire clinical team on the wards.

Additional ICD-10 diagnoses, other than SM or SAD, were classified into the following categories:

- internalizing disorders: mood (affective) disorders (ICD-10 F32-F34); neurotic, stress-related and somatoform disorders (ICD-10 F40-F48, excluding F40.1); emotional disorders with onset specific to childhood (ICD-10 F93.0 and F93.1)
- externalizing disorders: hyperkinetic disorders (ICD-10 F90); conduct disorders (ICD-10 F91); mixed disorders of conduct and emotions (ICD-10 F92)
- specific developmental disorders (ICD-10 F80-F83, F85-F89)
- other: any other additional ICD-10 diagnoses

##### Speaking behaviour at discharge

For the SM group, changes in speaking behaviour at discharge were categorized into three groups: ‘improved,’ ‘unchanged/unknown,’ or ‘worsened.’ This categorization was performed by the first author based on a structured review of the discharge summaries. Specifically, qualitative descriptions in the clinical documentation were screened for statements indicating improvement or deterioration in verbal communication. No standardized assessment tool was used, and the level of detail in the discharge notes varied, ranging from brief comments to more elaborate observations across different settings (e.g., school, ward, family).

##### Pharmacological compounds

Pharmacological compounds were classified into the following categories: SSRIs (including fluoxetine, sertraline, citalopram, escitalopram), non-SSRI antidepressants, antipsychotics, ADHD medication, benzodiazepines and melatonin preparations. The use of melatonin, as sleep-related medication, was included for completeness of the pharmacological treatment overview. 

Data extracted on SSRIs: initiation/discontinuation date, type of drug, initiation dosage, maximum dosage (per kilogram (kg) bodyweight). To compare dosages between SSRIs, equivalent doses for fluoxetine were determined based on clinical experience, established practice patterns, and existing evidence on recommended SSRI prescription practices in adults [35]. Fluoxetine was chosen as dosage equivalent standard, as it is a representative SSRI in terms of efficacy, tolerability, and potential adverse effects, additionally approved for the treatment of depressive disorders in children and adolescents. Accordingly, the equivalent dose was determined as follows: 1 mg fluoxetine per kg body weight = 5 mg sertraline per kg bodyweight = 1 mg citalopram per kg bodyweight = 0.5 mg escitalopram per kg bodyweight.

#### Statistical analysis

Statistical analysis was performed with R Software, version 4.2.2. For patients with more than one case, only the first case was included. Descriptive statistics, including measures of central tendency, variability, and distribution, were calculated using the *psych* and *compareGroups* packages. To account for potential confounding factors, the SM and SAD groups were matched on age at admission, sex, and year of admission using propensity score matching, implemented via the *MatchIt* package. Matching was performed using the nearest neighbour method with a 1:2 ratio without replacement. Propensity scores were calculated with a generalized linear model (GLM) as the distance measure. This matching approach ensured balanced baseline characteristics between the groups for the three matching variables. Descriptive data were compared between groups by t-tests for continuous variables and chi^2^ tests for categorical variables.

SSRI effects between groups were compared by linear regression models, first, evaluating the interaction and main effects of group (SM/SAD) and SSRI use (yes/no), and, second, assessing the interaction and main effects of SSRI dosage (fluoxetine-equivalent dosage per kg body weight) and group. Models were adjusted for age at admission, sex, and the number of additional internalizing, externalizing, and developmental disorders. To investigate speaking pattern in the SM group, logistic regression models were used, with the outcome categorized as “improved” versus the combined categories “worsened” and “not improved/unknown”. Independent variables included SSRI use (yes/no) and the fluoxetine-equivalent dosage (mg/kg). These models were also adjusted for age at admission, sex, and the number of additional internalizing, externalizing, and developmental disorders. □ was set at 0.05, indicating a significant difference between groups or SSRI use, without adjustment for multiple testing. 

## Results

### Sample characteristics

The final study population characteristics are summarized in Table 1. The sample consisted of 169 participants, divided into the SM group (*N* = 69) and the SAD group (*N* = 100). Both groups had a predominance of female participants, with 56.5% in the SM and 59.0% in the SAD group. The mean age at admission was 11.9 years in both groups. Results of IQ assessments were available for 113 participants. As expected due to the matching procedure, there were no statistically significant differences in IQ scores between the SM and SAD groups. Regarding additional mental disorders, internalizing disorders were most common in both groups, followed by specific developmental and externalizing disorders. SM individuals showed a lower rate of additional internalizing (*p* = 0.001) and externalizing (*p* = 0.058) disorders than SAD. SSRI treatment was more frequent in SM (75.4%) than in SAD (55.0%). Other psychopharmacological therapies were prescribed to 11.6% of participants in the SM group and 29% in the SAD group, either alongside or independently of SSRI treatment. This difference was largely due to a higher rate of ADHD medication in the SAD group (18.0% vs. 5.8%). Melatonin preparations were prescribed more often in the SM group than in the SAD group (8.7% vs. 3.0%).

**Table 1:** Sample characteristics

	SM (*N* = 69)	SAD (*N* = 100)
Age at admission (mean [years] [SD])	11.9 (3.63)	11.9 (2.59)
Sex (females, *n* [%])	39 (56.5)	59 (59.0)
Non-verbal IQ (*n* | mean [SD])	46 | 95.9 (13.8)	67 | 104.2 (14.3)
Verbal comprehension (*n* | mean [SD])	18 | 101.0 (20.5)	59 | 101.0 (14.3)
Number of additional internalizing disorders (mean [SD])	1.06 (1.07)	1.87 (1.18)
SAD as comorbidity (*n* [%])	62 (89.9)	
Number of comorbid externalizing disorders (mean [SD])	0.19 (0.52)	0.36 (0.64)
Number of comorbid specific developmental disorders (mean [SD])	0.39 (0.62)	0.43 (0.78)
Duration of treatment (mean [days] [SD])	139 (66.3)	120 (87.4)
Any SSRI medication (*n* [%])	52 (75.4)	55 (55.0)
Switch of SSRI medication (*n* [%])	5 (9.6)	3 (5.5)
Other non-SSRI antidepressants (*n* [%])	0 (0.0)	3 (3.0)
Antipsychotics (*n* [%])	3 (4.35)	7 (7.0)
ADHD medication (*n* [%])	4 (5.8)	18 (18.0)
Benzodiazepines (*n* [%])	1 (1.45)	1 (1.0)
Nutritional supplements (*n* [%])	3 (4.35)	4 (4.0)
Analgesics (*n* [%])	0 (0.0)	0 (0.0)
Antiallergic medications (*n* [%])	0 (0.0)	1 (1.0)
Melatonin preparations (*n* [%])	6 (8.7)	3 (3.0)
Antibiotics (*n* [%])	0 (0.0)	1 (1.0)
Other medications (*n* [%])	0 (0.0)	0 (0.0)

*SM* selective mutism

*SAD* social anxiety disorder

*N* number of individuals in full sample

*n* number of individuals in subsample

*SSRI* selective serotonin reuptake inhibitor

*ADHD* attention deficit hyperactivity disorder

### SSRI use in SM and SAD

Table 2 details the mean dosages and frequency of specific SSRIs among participants receiving any SSRI. In the SM group, escitalopram was the most commonly prescribed SSRI (60.8%), followed by citalopram (25.5%), sertraline (9.8%), and fluoxetine (3.9%). Similarly, escitalopram was also the most frequently prescribed SSRI in the SAD group (41.8%), followed by citalopram (25.5%), sertraline (20%), and fluoxetine (12.7%). Escitalopram was prescribed more frequently in the SM group compared to the SAD group. of sertraline, citalopram, and escitalopram were descriptively higher in the SM group than in the SAD group; however, these differences did not reach statistical significance (*p*_all_ > 0.05). Fluoxetine equivalent dosages also showed no differences between groups, with 0.65 mg/kg (SD 0.29) in the SM group and 0.61 mg/kg (SD 0.33) in the SAD group (*p* = 0.597).

**Table 2:** SSRI-treatment: frequency and dosage in SM and SAD

	SM (*N* = 51#)	SAD (*N* = 55)
Fluoxetine (*n* [%] | mean [mg/kg] [SD])	2 (3.9) | 0.52 (0.05)	7 (12.7) | 0.52 (0.25)
Sertraline (*n* [%] | mean [mg/kg] [SD])	5 (9.8) | 2.42 (0.98)	11 (20) | 1.85 (1.09)
Citalopram (*n* [%] | mean [mg/kg] [SD])	13 (25.5) | 0.58 (0.21)	14 (25.5) | 0.52 (0.13)
Escitalopram (*n* [%] | mean [mg/kg] [SD])	31 (60.8) | 0.3 (0.09)	23 (41.8) | 0.25 (0.09)
Fluoxetine equivalence dosage [mg/kg] (SD)	0.65 (0.29)	0.61 (0.33)

*SSRI* selective serotonin reuptake inhibitor

*SM* selective mutism

*SAD* social anxiety disorder

^#^ weight information missing in one individual

*N* total number of individuals receiving any SSRI

*n* number of individuals receiving a specific SSRI

*mg* milligram

*kg* kilogram

### SSRI switch and discontinuation

**Fig. 1:**
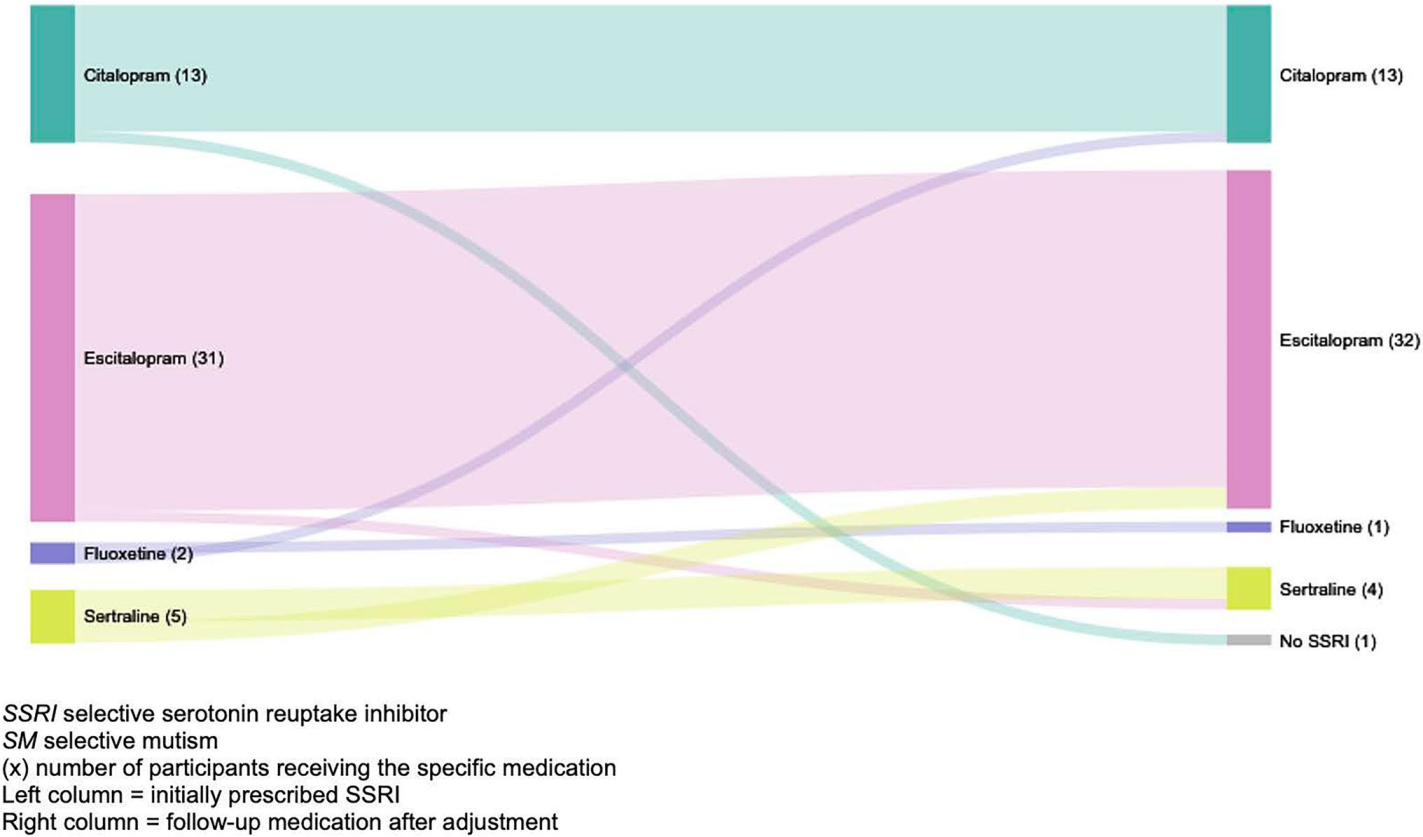
SSRI adjustments in SM cohort

**Fig. 2:**
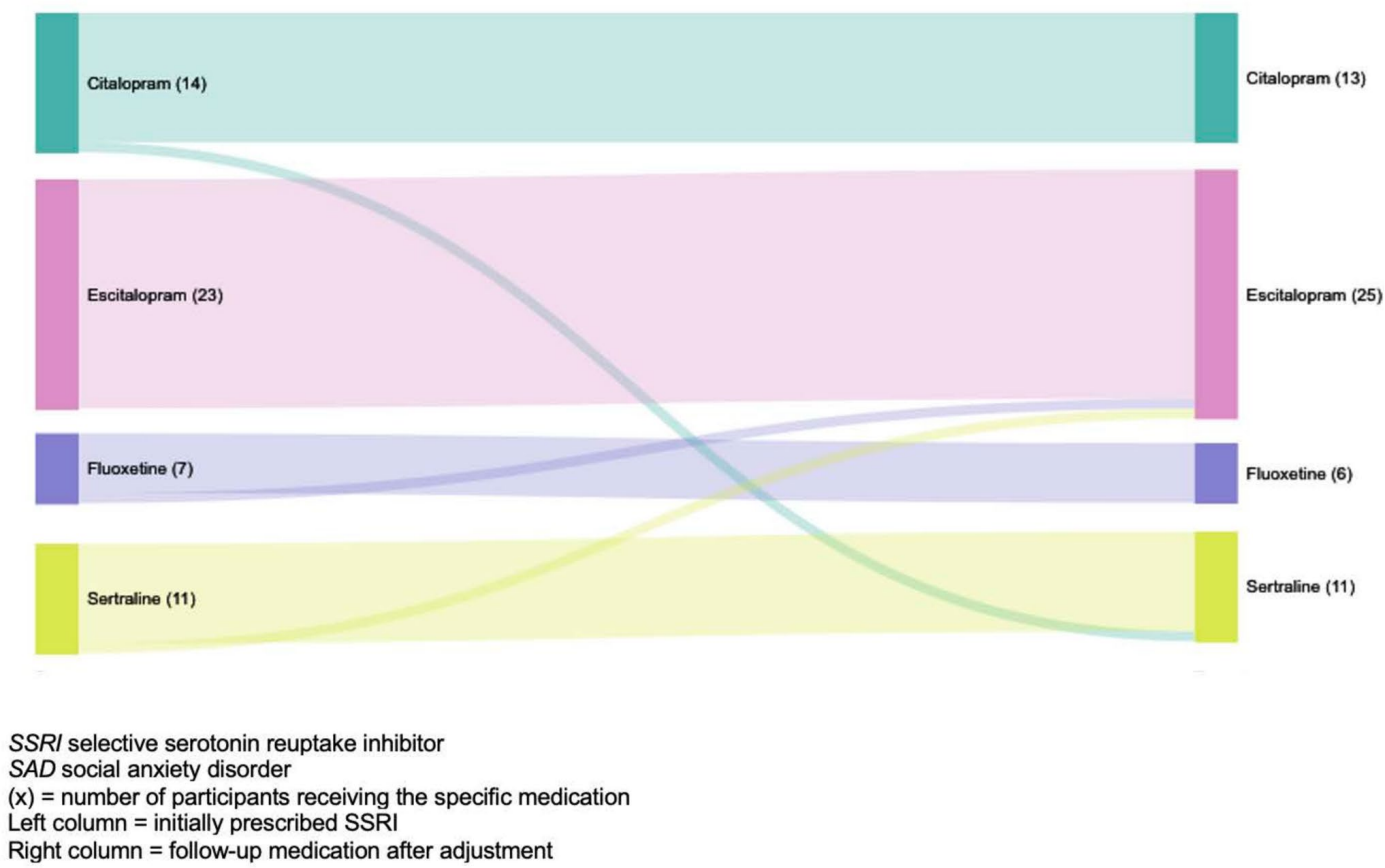
SSRI adjustments in SAD cohort

As shown in Figs. 1 and 2, only eight patients (five in the SM and three in the SAD group) experienced an SSRI switch or discontinuation during treatment. No statistically significant group differences were observed.

### Duration of inpatient/day-care treatment

DOT was descriptively slightly longer in the SM group (mean 139 days, SD 66.3) than in the SAD group (mean 120 days, SD 87.4). In both groups, Individuals treated with SSRIs, descriptively showed a longer DOT compared to those not receiving SSRIs (see Table 3). Comparing groups by linear regression adjusted for age at admission, sex, number of internalizing, externalizing and specific developmental disorders, no main or interaction effects of SSRI treatment and group were detected (*p*_all_ > 0.05). Number of externalizing disorders correlated positively with inpatient/day-care treatment duration across groups (β = 29.1, SE = 11.0, *p* = 0.009). Similarly, no main or interaction effects of SSRI dosage and group were observed (*p*_all_ > 0.05).

**Table 3:** Influence of SSRI medication on treatment duration

	Duration of treatment in days		Statistics#		
	SM (*N* = 69)	SAD (*N* = 100)	Main effect of group	Main effect of SSRI	Interaction group x SSRI
With SSRI (*n* | mean DOT [SD])	52 | 143 (58.2)	55 | 133 (108)	β = −27.6 SE = 23.5 *t* = −1.17 *p* = 0.242	β = 13.6 SE = 23.2 *t* = 0.59 *p* = 0.559	β = 11.1 SE = 27.6 *t* = 0.40 *p* = 0.688
Without SSRI (*n* | mean DOT [SD])	17 | 124 (87.1)	45 | 104 (48.5)			

*DOT* duration of treatment [days]

*SSRI* selective serotonin reuptake inhibitor

*SM* selective mutism

*SAD* social anxiety disorder

*N* total number of individuals

*n* number of individuals in the subsample

^#^ Linear regression model adjusted for: age at admission, sex, number of additional internalizing/externalizing/developmental disorders

### Speaking behaviour at discharge (SM group)

Speaking pattern at discharge is shown in Table 4. Improved speaking behaviour was more frequently observed in participants in the SSRI-treated subgroup (*n* = 45, 93.8%) compared to the subgroup not receiving SSRI treatment (*n* = 10, 62.5%). Logistic regression, adjusted for age, sex, number of internalizing, externalizing and specific developmental disorders showed a strong influence of SSRI treatment on improved speaking pattern (β = 2.7, SE = 1.1, *p* = 0.009), whereas number of internalizing disorders were correlated with a lower odds of improved speaking (β = −1.4, SE = 0.5, *p* = 0.012).

**Table 4:** Children with selective mutism: comparison of speaking pattern at discharge by SSRI treatment

	no SSRI (*N* = 16)	any SSRI (*N* = 48)	Statistics#
Improved (*n* [%])	10 (62.5)	45 (93.8)	β = 2.75 SE = 1.06 z = 2.60 *p* = 0.009
Worsened (*n* [%])	1 (6.25)	0	
Not changed/unknown (*n* [%])	5 (31.25)	3 (6.25)	

*SSRI* selective serotonin reuptake inhibitor

*N* number of individuals in full sample

*n* number of individuals in subsample

^#^ Linear regression model adjusted for: age at admission, sex, number of additional internalizing/externalizing/developmental disorders

## Discussion

To gain more insight into the effectiveness of SSRI treatment in routine clinical care for severely affected children with mental disorders, we described SSRI prescription patterns, and compared the effect of SSRI treatment on the DOT in a large day-care/inpatient sample of children with SM and SAD. We hypothesized that SSRI treatment was associated with lower DOT in both groups. Additionally, for the SM group, we hypothesized a more strongly improved speaking pattern for individuals receiving SSRIs compared to those without SSRI treatment.

Consistent with existing epidemiological data on SM and SAD, individuals included in our study were predominantly female [6]. Mean age at admission was 11.9 years in both groups, which contrasts with the typical early childhood onset of SM [1], and is slightly younger than the average SAD onset [36]. Individuals in our study were treated as day-care or inpatients, thus, all children showed a rather severe expression of SM and SAD, which had not been sufficiently improved by outpatient intervention. Due to the early onset of SM, it is likely, that most children included in our sample showed a chronic course of SM, which may also influence intervention effects [37]. Importantly, previous research has shown that early intervention is crucial for treatment success in SM: younger children (aged 3–5 years) show significantly better outcomes than older children (aged 6–9 years), with higher remission rates and more substantial symptom reduction. These findings underline the importance of early diagnosis and timely treatment initiation to improve long-term outcomes in SM [2, 2, 29, 29].

In addition, most children showed additional internalizing, externalizing or specific developmental disorders, indicative of a high (poly-)genetic load for mental disorders in these children [38]. Children with SAD even showed a higher rate of additional internalizing and externalizing disorders than those with SM. As the SAD group was selected based on matching the SM group with regard to age and sex, it differs from epidemiological and many clinical studies on SAD, which reported lower rates of externalizing disorders and a slightly older average age of onset [39–42]. Consistent with previous studies in SM, around 90% of participants in our SM group were diagnosed with comorbid SAD [14, 39]. Also, the children with SM in our sample with available IQ data showed average non-verbal cognitive abilities similar to children with SM included in other studies. Taken together, as we studied individuals with SM and SAD who were treated as day-care or inpatients, our results pertain to a population of severely affected individuals with additional comorbid mental disorders.

Given that the mean age in our SM group was 11.9 years—substantially older than the typical onset age of SM [2]—the findings should be interpreted in the context of a more chronic and potentially treatment-resistant population. The inpatient and day-care setting is likely to reflect a subset of SM patients with greater symptom severity, higher comorbidity, and prior insufficient response to outpatient interventions. Moreover, the multimodal treatment provided, combining intensive CBT, speech and language therapy, and pharmacotherapy, is less commonly available in routine outpatient care. In outpatient settings, particularly for younger children, SSRIs may be prescribed less frequently and at lower dosages than observed in our study. Therefore, while our results provide important insights into SSRI treatment in SM, generalization to younger, outpatient populations should be made with caution, and future research is needed to determine whether similar effects are observed in these groups.

The high rate of SSRI use in both groups likely reflects the chronicity and severity of the disorders, with the even higher prescription rate in SM possibly attributable to its persistent course and frequent comorbidity with SAD.

In our study, escitalopram was the most frequently prescribed SSRI in both groups, particularly in SM, despite fluoxetine and sertraline being more prominently recommended in guidelines and research for pediatric anxiety disorders, including SM [15]. Our findings provide some clinical evidence for the use of escitalopram, citalopram, and sertraline in SM; however, this evidence is derived from older school-aged children, in contrast to the younger age at which SM typically manifests [14]. The preference for escitalopram may reflect institutional prescribing practices, perceived tolerability, and clinician familiarity, rather than evidence-based prioritization. Due to the low number of children treated with some SSRIs, a direct efficacy comparison was not feasible. Instead, we compared dosages and fluoxetine-equivalent doses between SM and SAD, which showed no significant differences.

For fluoxetine, the mean dosage in our SM group (0.52 mg/kg/day, SD 0.05) was slightly lower than the upper range reported in the literature for SM (up to 0.6 mg/kg/day) [23, 43] yet consistent with prior recommendations [44]. In SAD, Birmaher et al. [45] suggested 20 mg/day in a randomized, placebo-controlled trial for youths aged 7–17 years. For escitalopram, the mean dosage in SM (0.30 mg/kg/day, SD 0.09) was slightly higher than in SAD (0.25 mg/kg/day, SD 0.09). While only case series are available for its use in SM [26] and no dosing recommendations exist for this group, the dosages in our study are broadly in line with those reported for pediatric anxiety disorders [44]. For sertraline, our observed dosages in SM (129 mg/day, SD 69.7) were within the range reported in RCTs on SM and other anxiety disorders [24, 44, 44, 46, 46]. In a double-blind trial involving five children with SM, a positive effect was observed at 100 mg/day [24]. In an RCT on various anxiety disorders, Walkup et al. [46] reported improvements in 54.9% of participants receiving sertraline alone (mean 146 mg/day), in 80.7% receiving sertraline + CBT (mean 133.7 mg/day), and in 59.7% receiving CBT only. Similar to escitalopram, no studies investigating citalopram use in SM are currently available, and no specific research has examined its use for pediatric SAD [15, 47]. Varia et al., through case reports, suggested potential benefits for adult SAD [48]; however, evidence on pediatric dosing remains limited. Nonetheless, our citalopram dosages align with recommendations for pediatric anxiety disorders in general [44, 49]. Given that CBT was systematically integrated into multimodal treatment for all patients, our dosage patterns are unlikely to be explained by concurrent CBT administration. Other factors—such as the inpatient/day-care setting, close clinical monitoring, and patient-specific treatment planning—may have contributed to the observed prescribing patterns.

Beyond SSRI treatment, patients in the SAD group were more frequently prescribed additional psychopharmacological medications. This may reflect greater clinical complexity in terms of behavioural difficulties, attentional problems, or comorbidities requiring pharmacological management. In contrast, melatonin was prescribed more often in the SM group (8.7% vs. 3.0%), possibly indicating increased challenges related to sleep initiation or regulation in this subgroup. However, as melatonin was prescribed only in a minority of cases, this cannot be taken as evidence of generally preserved sleep quality. Due to the retrospective nature of the study, sleep problems were not assessed systematically and cannot be reliably inferred from medication data alone. Furthermore, the specific indications for the use of medications—particularly for antipsychotics—were not systematically documented and therefore could not be analyzed.

Contrary to our hypothesis, SSRI treatment did not reduce DOT in either group, and neither the type of SSRI nor its dosage significantly influenced DOT. To date, no studies have specifically investigated the length of inpatient or day-care treatment in SM, particularly in relation to the impact of SSRI use or additional mental disorders. Future studies should investigate the role of comorbidities, as externalizing disorders in our study were positively correlated with DOT. Furthermore, DOT in both groups exceeded the average length of inpatient care in child and adolescent psychiatric clinics in Germany [32], again reflecting disorder severity and chronicity [39], presumably related to high (poly-) genetic load [38].

In our study, DOT refers to the duration of inpatient or day-care treatment rather than the duration of SSRI administration. According to current guidelines [50], SSRI treatment should be maintained for at least 12 months after remission. At discharge, recommendations are provided in line with these guidelines. Outpatient follow-up is typically conducted by child and adolescent psychiatrists and therapists, who monitor clinical status and evaluate the ongoing need for medication. For the patients included in this study, post-inpatient care was partly provided at the outpatient clinic of the Department of Psychiatry, Psychosomatics and Psychotherapy of Childhood and Adolescence, University Hospital Frankfurt, whereas most continued treatment in the community, particularly regarding psychotherapeutic follow-up.

In line with our hypothesis, SSRI use was significantly associated with greater improvements in speaking behaviour among SM patients. While the efficacy of SSRIs in ameliorating SM symptoms has been studied, specific research focusing on their direct impact on speaking behaviour remains scarce, particularly in comparison between medicated and unmedicated groups. Existing studies, mostly with small sample sizes, have typically reported general symptom improvements, such as reductions in anxiety, increased verbal output, and enhanced global functioning based on clinician or parental ratings [15, 51]. The heterogeneity of measurement instruments in prior studies further complicates cross-study comparisons [43]. However, caution is warranted when interpreting our findings. In our study, speaking behaviour at discharge was assessed from clinical documentation, and improvements observed during treatment may not necessarily generalize to other settings such as school, public situations, or interactions outside the hospital.

Furthermore, clinicians may be inclined to rate discharge status optimistically. These factors may have contributed to the observed association and underline the need for standardized, validated measures in future research, such as the Frankfurt Scale for the Assessment of Selective Mutism (FSSM) [52]. Our findings also suggest that comorbidities, particularly additional internalizing disorders, may adversely affect improvements in speaking behaviour. This highlights the importance of accounting for such conditions in both research and clinical practice to optimize therapeutic approaches for SM [14, 39].

### Limitations

This study has several limitations. As data were collected from a single department of child and adolescence psychiatry, psychosomatics and psychotherapy, both the sample size and associated statistical power are limited. Expanding the study to a multicenter design, younger age groups, or incorporating data from outpatient departments may increase the sample size and statistical power but would also introduce greater clinical heterogeneity.

Given the retrospective nature of the study, we were limited to routinely collected clinical data and information extractable from discharge letters. Therefore, we could not control for additional covariates that may have influenced DOT or clinical outcomes, such as parental psychopathology and socioeconomic status [53]. Moreover, the study design did not allow for a systematic analysis of which specific components of the multimodal treatment were associated with clinical improvement. While all patients received care within an integrated treatment framework, including individualised tailored CBT, group therapy, pharmacotherapy, and, for SM, additionally speech and language therapy, the intensity, duration, and specific content of these interventions were not consistently documented and may have varied over the 10-year study period. As a result, it remains unclear whether improvements, particularly in speaking behaviour, can be attributed to specific interventions or to the broader therapeutic context.

Although all diagnoses were made within a standardized and well-established diagnostic framework – incorporating clinical interviews, behavioural observations, and validated questionnaires—the FSSM [52] was not consistently applied across all patients. While diagnostic procedures were conducted by trained professionals according to clinical best practice, the absence of uniform use of standardized instruments may limit the reliability and comparability of specific diagnoses, particularly differentiating SM from other anxiety disorders.

In addition, the categorization of speaking behaviour at discharge was based on a retrospective review of discharge letters and not on a standardized assessment tool. The level of detail in clinical documentation varied, and ratings were based on the clinical judgment of the first author. This approach may have introduced subjectivity and limited the comparability across patients. Furthermore, information on the indications for non-SSRI psychopharmacological treatment were not systematically documented and therefore could not be analyzed. Furthermore, improvements observed during inpatient or day-care treatment may not necessarily generalize to other settings, such as school, home, or public situations, and clinicians may tend to adopt an optimistic perspective when evaluating discharge status.

Finally, the SM group in our study consisted of older school-aged children and adolescents, whereas SM typically manifests in early childhood [2]. This age difference likely reflects a more chronic and potentially treatment-resistant subgroup, often with higher comorbidity and a history of insufficient response to outpatient interventions. This also highlights the potential impact on the generalizability of our findings to younger children. The day-care and inpatient setting of our study provided an intensive multimodal treatment not possible during outpatient care, which may limit the applicability of our findings to younger, less severely affected SM populations.

### Conclusions

To our knowledge, this study is the first to study effectiveness of SSRI treatment in routine clinical care in patients with SM compared to those with SAD. Our findings enhance the understanding of pharmacological treatment for SM, and highlight directions for future research. Multicenter randomized controlled trials (RCTs) with adequate statistical power are needed to expand the evidence base for SM treatment, ideally combining pharmacological and psychotherapeutic interventions. Future studies should also apply standardized, disorder-specific assessment tools and include sensitive outcome measures that can capture meaningful clinical change. Importantly, future research should aim to disentangle the specific effects of individual therapeutic components within multimodal interventions. This includes examining the relative contribution of speech and language therapy, individual CBT (e.g., exposure techniques), and group interventions to treatment outcomes. Such analyses could inform more targeted, efficient treatment protocols.

In addition, future studies should explicitly include severely affected individuals with multiple psychiatric comorbidities, and examine the impact of internalizing, externalizing, and developmental disorders on treatment outcomes. These factors likely contribute to treatment complexity and may moderate or mediate intervention effects.

In line with previous findings, early identification and intervention are crucial in preventing chronic courses of SM and improving long-term outcomes. Several studies – as named above—have shown that early treatment, especially when combining evidence-based psychotherapeutic strategies, can significantly enhance treatment response and reduce the risk of symptom persistence.

Despite the absence of approved medications for SM in pediatric populations, SSRIs are frequently used based on their established efficacy in treating other anxiety disorders in children and youths. Clinicians should flexibly incorporate SSRIs into a multimodal intervention approach, ensuring informed consent and close monitoring of efficacy and tolerability. The notably high use of escitalopram compared with guideline-recommended SSRIs (fluoxetine, sertraline) observed in this study highlights the need for further investigation of prescribing practices and treatment selection in SM. Individualized treatment plans remain essential to address the heterogeneity of symptoms.

Overall, our findings emphasize the need for further research to expand the evidence base, refine treatment protocols, and improve outcomes for children and youths with SM.

## Data Availability

Anonymised data are available to other researchers upon request.
